# In Vivo Antioxidant and Nephroprotective Effects of Ethanolic Extract of Carica papaya Seeds and Its Isolated Flavonoid on Gentamicin-Induced Nephrotoxicity in Wistar Albino Rats

**DOI:** 10.7759/cureus.57947

**Published:** 2024-04-10

**Authors:** Sarikonda Sandhya Rani, T Vedavijaya, Karuna Sree Podila, Zubair Ahmed Md, Soujanya Chinnanolla, Suresh Babu Sayana

**Affiliations:** 1 Department of Pharmacology, Meenakshi Academy of Higher Education and Research, Chennai, IND; 2 Department of Pharmacology, Meenakshi Ammal Dental College and Hospital, Chennai, IND; 3 Department of Pharmacology and Therapeutics, All India Institute of Medical Sciences, Kalyani, Kalyani, IND; 4 Department of Pharmacology, Mamata Academy of Medical Sciences, Hyderabad, IND; 5 Department of Regulatory Toxicology, National Institute of Pharmaceutical Education and Research, Hyderabad, IND; 6 Department of Pharmacology, Government Medical College and General Hospital, Suryapet, IND

**Keywords:** nephroprotective, histopathological analysis, oxidative stress, renal function, wistar albino rats, antioxidant, isoliquiritigenin, carica papaya seeds, nephrotoxicity, gentamicin

## Abstract

Background

The nephrotoxic side effects of gentamicin, a potent aminoglycoside antibiotic, significantly restrict its clinical use. Identifying compounds that can mitigate this nephrotoxicity is of paramount importance. The research examines how the ethanolic extract of *Carica papaya* seeds (EECPS) and isoliquiritigenin (ISL), a flavonoid separated from them, protect the kidneys and fight free radicals in gentamicin-treated Wistar albino rats.

Methodology

A total of 48 mature Wistar albino rats were divided into eight groups, with each group consisting of six rats. The experimental setup included a normal control group receiving oral saline as a negative control, and a standard control group administered gentamicin intraperitoneally (IP) at 100 mg/kg body weight for 13 days to induce nephrotoxicity, followed by oral silymarin at 100 mg/kg body weight as a positive control from days 14 to 21. A toxicant control group was exposed to gentamicin IP without subsequent treatment. Two test groups were given 400 mg/kg and 800 mg/kg of EECPS orally after being given gentamicin. Three other test groups were given 20 mg/kg, 40 mg/kg, and 80 mg/kg of ISL orally after being given gentamicin. Serum levels of creatinine, urea, and blood urea nitrogen (BUN) were used to test renal function. Malondialdehyde (MDA), nitric oxide (NO), and reduced glutathione (GSH), which are signs of oxidative stress, were also measured in renal tissues.

Results

Gentamicin administration markedly increased serum creatinine, urea, and BUN levels, confirming its nephrotoxic effect. Nephroprotection depended on the dose of EECPS and ISL used. It was found that 80 mg/kg of ISL had the most powerful effect, which was not what was thought at first. These treatments effectively reduced MDA and NO levels while enhancing GSH levels, exhibiting their strong antioxidant properties. Notably, the nephroprotective efficacy of these treatments exceeded that of silymarin, a known nephroprotective agent. Histopathological analysis confirmed reduced renal damage and enhanced tissue repair in the treated groups.

Conclusions

These findings demonstrate how effective EECPS and ISL are at shielding the kidneys from gentamicin-caused damage. They do this by acting as antioxidants and nephroprotectants. Their ability to protect kidney function and fight oxidative stress makes them interesting as possible treatments for gentamicin-related kidney damage. These results advocate for further investigation into the utility of these natural compounds in the management of nephrotoxicity.

## Introduction

The pervasive use of aminoglycoside antibiotics, especially gentamicin, highlights a critical paradox in contemporary medical practice. Gentamicin’s robust antibacterial prowess, capable of combating a broad spectrum of pathogens, including those resistant to multiple drugs, is a testament to its invaluable contribution to infectious disease management [1]. However, nephrotoxicity poses a significant obstacle that overshadows its therapeutic value. Nephrotoxicity, which results in acute kidney damage due to oxidative stress, inflammation, and renal cell death, is a common side effect of gentamicin. This adverse outcome not only compromises patient health but also limits the clinical application of an otherwise effective antibiotic regimen [2]. Consequently, the development of effective nephroprotective strategies that can mitigate these side effects without detracting from the antibiotic’s efficacy is a pressing priority in the realm of patient care and therapeutic outcomes.

The *Carica papaya*, a tropical fruit known for its delicious taste and high nutritional value, has been suggested as a possible source of therapeutic agents in the field of phytotherapy [3]. As a result, the search for these nephroprotective compounds has grown. *Carica papaya* seeds have long been utilized in diverse civilizations for their therapeutic attributes, effectively treating a wide range of health conditions, including digestive disorders and parasite infections [4]. Scientific inquiry into these seeds has unveiled a rich phytochemical landscape, teeming with bioactive compounds such as flavonoids, alkaloids, and saponins. These constituents are heralded for their antioxidant, antimicrobial, and anti-inflammatory properties, making *Carica papaya* seeds a focal point of research into natural health solutions [5].

Isoliquiritigenin (ISL), a flavonoid found in *Carica papaya* seeds and licorice roots, has attracted interest due to its notable antioxidant and anti-inflammatory properties [6]. ISL is important because it can change biochemical pathways involved in oxidative stress and inflammation. These are two main factors in how gentamicin damages the kidneys [7,8]. *Carica papaya* seeds, and ISL in particular, are good sources of antioxidants that could help fight free radicals and lessen the damage they do to cells [9,10].

This study aims to determine how well ethanolic extract of *Carica papaya* seeds (EECPS) and ISL protect the kidneys from gentamicin-induced nephrotoxicity and how well they work as antioxidants. This study examines how these organic compounds might aid in gentamicin-induced kidney damage repair. The goal is to show that they could be used to prevent or reduce the effects of drug-induced nephrotoxicity. By doing this, it hopes to increase the therapeutic usefulness of gentamicin by protecting against its nephrotoxic side effects. This will make the antibiotic more clinically effective and improve patient outcomes. The exploration of these natural compounds not only contributes to the growing field of nephroprotective phytotherapy but also highlights the importance of integrating traditional medicinal knowledge with modern clinical practices to combat the challenges of drug-induced nephrotoxicity.

## Materials and methods

Location and duration of study

This research was performed at the Mamata Academy of Medical Sciences in Hyderabad, India. The study was conducted from January 2022 to June 2022, a duration of six months.

Seed gathering

Fresh *Carica papaya* seeds were extracted methodically from mature fruits. These fruits were grown in personal gardens, ensuring the selection of viable and completely developed seeds. Dr. P. Suresh Babu, Assistant Professor in the Department of Botany at Government Degree College, Kukatpally, Hyderabad, India, verified the authenticity of the *Carica papaya* seeds.

Cleaning and de-pulping

The gathered seeds were carefully cleaned to remove any remaining fruit pulp and unnecessary debris. This process ensured the purity of the seeds for subsequent analyses.

Drying process

The cleaned seeds were evenly spread out and exposed to regulated air-drying conditions to reach the required moisture content. The goal of this drying procedure was for the seeds to be as dry as possible.

Powder preparation

After thorough drying, the seeds were finely ground into a powder with a laboratory grinder. This procedure improved the efficiency with which bioactive chemicals were extracted from the seeds. The powder was kept at 4°C until further use in tight aliquoted vials.

Preparation of EECPS

Ethanol was chosen for Soxhlet extraction of *Carica papaya* seeds due to its high solubility in a variety of plant-based chemicals. The Soxhlet apparatus was used to extract the desired components from the ground seeds. The seeds were placed within a thimble and then placed inside the apparatus. As the ethanol was heated, it underwent vaporization, permeated the seed material, and subsequently underwent condensation back into a liquid. This process was repeated over several hours to achieve the maximum extraction of the bioactive components included in the seeds. After the extraction process, the ethanol solution was concentrated to remove the solvent, resulting in a crude ethanolic extract that contains a high number of bioactive compounds obtained from *Carica papaya* seeds. The soxhlet extraction method produced an ethanolic extract that functioned as the basis for further experimental studies. The extract was dissolved in sterile distilled water and kept in a cool, dark environment until needed for further experimental procedures.

Isolation of ISL

**Figure 1:**
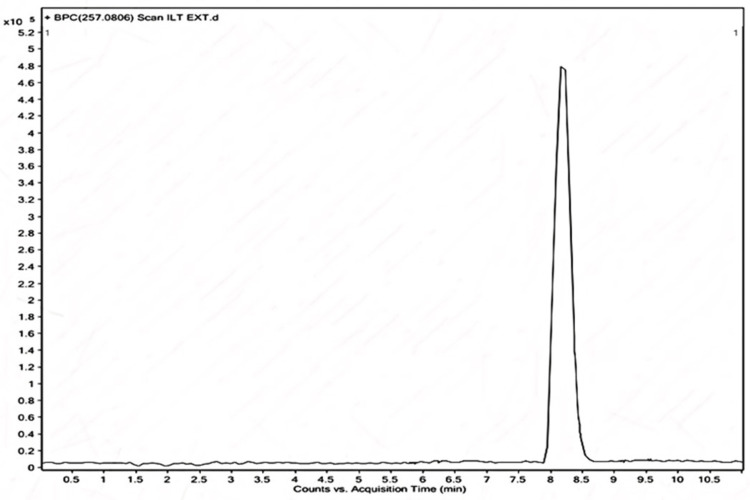
Quantitative phytochemical analysis: Isoliquiritigenin in ethanolic extract of Carica papaya seed using LC-MS (base peak chromatogram of extract (ILT-EXT). LC-MS = liquid chromatography-mass spectrometry

The flavonoid ISL in the EECPS was determined using high-resolution mass spectrometry (HRMS). The analysis utilized the following two test samples: ILT-STD (standard) and ILT-EXT (extract), employing chemicals and reagents such as liquid chromatography-mass spectrometry (LC-MS)-grade formic acid, methanol, and water. The configuration consisted of a quadrupole time-of-flight G6540B HRMS from Agilent Technologies, USA, which was combined with an Agilent 1200 infinite series UHPLC from Agilent Technologies, California, USA. The Mass Hunter workstation software 11 was utilized for data gathering and processing (Figure 1).

To prepare the samples, aliquots were dissolved in methanol. The flavonoid was quantitatively estimated using liquid chromatography with a shim-pack C18 column as the stationary phase (dimensions: 150 x 4.6 mm, 5 μm) and a mobile phase consisting of solvent A (0.1% formic acid in water) and solvent B (methanol). This methodology enabled the accurate detection and measurement of ISL in EECPS, providing a dependable way to evaluate the purity and concentration of this particular flavonoid.

Animal model

The study utilized male Wistar albino rats as the animal model. These rats are commonly employed in toxicological research due to their well-defined metabolic processes, which closely resemble those of humans. This similarity makes them an excellent model for evaluating the nephroprotective properties of various substances. For this study, male Wistar albino rats weighing between 150 and 200 g were utilized. They were housed in clean polypropylene cages, maintained in a controlled lab setting with a temperature around 22°C, and exposed to a 12-hour cycle of light and darkness. These animals were given free access to standard food and water.

Experimental design

The design included eight groups of six Wistar albino rats with each group subjected to different treatment protocols to assess the protective effects of EECPS and its isolated flavonoid against gentamicin-induced nephrotoxicity.

Group 1 (Normal Control)

Rats received oral administration of normal saline. This group served as a negative control, indicating baseline health without any induced nephrotoxicity or treatment effects.

Group 2 (Standard Control)

Rats were administered gentamicin intraperitoneally (IP) at a dose of 100 mg/kg body weight from days 1 to 13 to induce nephrotoxicity. From days 14 to 21, rats received silymarin orally (PO) at 100 mg/kg body weight, serving as a positive control for nephroprotection.

Group 3 (Toxicant Control)

Rats received gentamicin IP at 100 mg/kg body weight for 1-13 days with no subsequent treatment. This group served to demonstrate the effects of nephrotoxicity without any protective intervention.

Group 4 (Low-Dose EECPS Treatment)

Rats were given gentamicin IP at 100 mg/kg body weight from days 1 to 13. From days 14 to 21, rats were treated with a lower dose (400 mg/kg body weight) of the EECPS orally.

Group 5 (High-Dose EECPS Treatment)

Rats were given gentamicin IP at 100 mg/kg body weight from days 1 to 13 and received a higher dose (800 mg/kg body weight) of EECPS orally from days 14 to 21.

Group 6 (ISL 20 mg/kg Treatment)

Rats in this group received gentamicin IP at 100mg/kg body weight from days 1 to 13 to induce nephrotoxicity. From days 14 to 21, these rats were treated with the isolated flavonoid, ISL, orally at a dose of 20 mg/kg body weight.

Group 7 (ISL 40 mg/kg Treatment)

Similar to Group 6, rats in Group 7 were administered gentamicin IP at 100 mg/kg body weight for the first 13 days to induce nephrotoxicity. From days 14 to 21, the treatment involved administering ISL orally at a mid-range dose of 40 mg/kg body weight.

Group 8 (ISL 80 mg/kg Treatment)

This group also underwent gentamicin IP administration at 100 mg/kg body weight from days 1 to 13 to induce nephrotoxicity. However, from days 14 to 21, the rats were treated with a high dose of ISL orally at 80 mg/kg body weight.

Evaluation of nephroprotective and antioxidant effects

To assess the nephroprotective effect, serum creatinine, urea, and blood urea nitrogen (BUN) levels were measured using standard biochemical methods after the treatment period. For antioxidant evaluation, the kidney tissues were homogenized, and the levels of oxidative stress markers, including malondialdehyde (MDA) for lipid peroxidation, nitric oxide (NO), and reduced glutathione (GSH), were quantified.

Statistical analysis

The results are shown as mean ± standard deviation. A one-way analysis of variance (ANOVA) was used to conduct the statistical analysis, followed by comparisons across groups using Dunnett’s post hoc test. The statistical significance was determined with a p-value below 0.05. The statistical evaluations were conducted using SPSS version 22.0 (IBM Corp., Armonk, NY, USA).

Ethical approval

The Institutional Animal Ethics Committee (IAEC) of Mamata Academy of Medical Sciences, located in Hyderabad, India, granted ethical approval for conducting animal experimentation (approval number: 01/MAMS/IAEC/2021).

## Results

Quantitative study of phytochemicals, specifically ISL, in EECPS seeds using LC-MS

The LC-MS analysis produced significant details regarding the molecular mass of ISL found in the EECPS. The precise molecular mass of ISL was identified, which is essential for its identification and quantification.

Purity of ISL

The ISL used in this study was confirmed to be 90.5% pure. This implies that this substance was responsible for the observed nephroprotective and antioxidant effects.

Elevated nephrotoxicity markers due to gentamicin

When gentamicin was given, serum levels of urea, creatinine, and BUN increased significantly, showing that the drug was harmful to the kidneys. Urea levels peaked at 355 ± 12.5 mg/dL, and BUN levels increased to 165.8 ± 8.3 mg/dL. These figures stand in stark contrast to those observed in the normal control group, where creatinine levels were 0.67 ± 0.03 mg/dL, urea levels were 48.4 ± 2.1 mg/dL, and BUN levels were 22.6 ± 1.3 mg/dL.

Nephroprotective effects of EECPS and ISL

**Figure 2:**
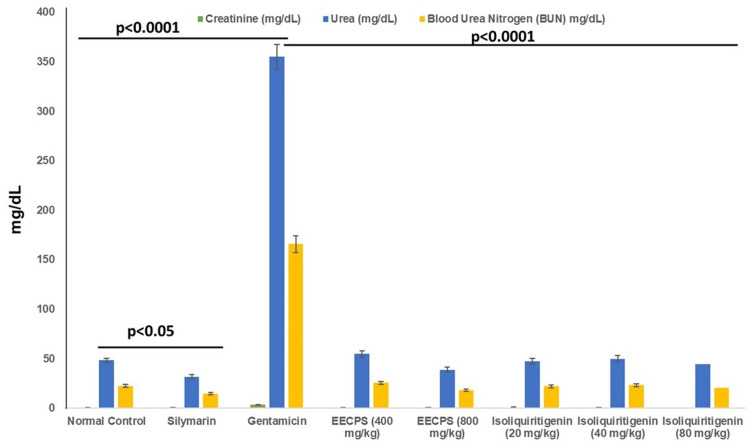
Comparative evaluation of the nephroprotective effects of EECPS (400 mg/kg and 800 mg/kg) and ISL (20 mg/kg, 40 mg/kg, and 80 mg/kg) on gentamicin-induced nephrotoxicity in Wistar albino rats. Normal creatinine: 0.4–0.8 mg/dL; normal urea: 15-45 mg/dL; normal BUN: 15-22 mg/dL. ANOVA was employed to compare the means of three or more groups. The Mann-Whitney U test was utilized to compare two independent groups. The Kruskal-Wallis test, a non-parametric counterpart of ANOVA, was used to compare three or more groups when the data was not normally distributed. Similarly, the Wilcoxon signed-rank test, a non-parametric equivalent of the paired t-test, was applied when the data did not follow a normal distribution. EECPS = ethanolic extract of Carica papaya seeds; ISL = isoliquiritigenin; BUN = blood urea nitrogen; ANOVA = analysis of variance

Treatment with EECPS demonstrated dose-dependent nephroprotective effects. At 400 mg/kg, EECPS reduced creatinine to 0.9 ± 0.05 mg/dL, and at 800 mg/kg, further decreased it to 0.56 ± 0.04 mg/dL. Urea and BUN levels also improved with EECPS treatment, dropping to 55 ± 3.2 mg/dL and 25.7 ± 1.6 mg/dL at the lower dose, and to 38.9 ± 2.8 mg/dL and 18.1 ± 1.2 mg/dL at the higher dose, respectively (Figure 2).

ISL treatment at 20 mg/kg, 40 mg/kg, and 80 mg/kg also showed dose-dependent reductions in nephrotoxicity markers. The most notable improvement was observed with the 80 mg/kg dose, where creatinine levels decreased dramatically to 0.33 ± 0.02 mg/dL, urea levels to 44.5 ± 2.5 mg/dL, and BUN levels to 20.7 ± 1.3 mg/dL. This suggests that the 80 mg/kg dose of ISL possesses potent nephroprotective properties.

Comparative efficacy with silymarin

Compared to the silymarin-treated group, which showed creatinine levels of 0.79 ± 0.04 mg/dL, urea levels of 31.9 ± 1.9 mg/dL, and BUN levels of 14.9 ± 1.1 mg/dL, the high-dose treatments of EECPS (800 mg/kg) and ISL (80 mg/kg) demonstrated superior efficacy in mitigating the biochemical markers of nephrotoxicity.

Antioxidant effects of EECPS and ISL on oxidative stress markers in kidney tissues of Wistar albino rats

The study assessed the antioxidant effects of EECPS and ISL on oxidative stress markers in the kidney tissues of Wistar albino rats. The parameters measured included MDA, NO, and GSH, which are critical indicators of lipid peroxidation, nitrosative stress, and antioxidant capacity, respectively.

MDA

The oxidative stress control group exhibited high MDA levels at 4.0 nM/mg protein, indicative of lipid peroxidation. However, the EECPS-treated group showed a significant reduction in MDA levels to 2.0 nM/mg protein, while the ISL-treated group further decreased MDA to 1.36 nM/mg protein, illustrating robust antioxidant effects.

NO

High NO levels were observed in the control group at 25 μM/mg protein, representing nitrosative stress. Treatment with EECPS resulted in a noteworthy decrease in NO levels to 15 μM/mg protein. The ISL-treated group exhibited a comparable reduction, with NO levels at 16.67 μM/mg protein.

GSH

GSH levels in the control group were found to be 10 μM/mg protein, reflecting a lower antioxidant capacity. In contrast, treatment with EECPS doubled the GSH levels to 20 μM/mg protein. ISL treatment displayed even higher efficacy in increasing GSH levels to 22.3 μM/mg protein (Table 1).

**Table 1:** Antioxidant effects of EECPS and ISL on oxidative stress markers in kidney tissues of Wistar albino rats. Control group: Standard control where the rats were administered gentamicin to induce nephrotoxicity where the oxidative stress was higher. * next to the values in the EECPS-treated group and ISL-treated group columns indicates that these values are statistically significant compared to the control group (oxidative stress). ANOVA was employed to compare the means of three or more groups. The Mann-Whitney U test was utilized to compare two independent groups. The Kruskal-Wallis test, a non-parametric counterpart of ANOVA, was used for comparing three or more groups when the data was not normally distributed. Similarly, the Wilcoxon signed-rank test, a non-parametric equivalent of the paired t-test, was applied when the data did not follow a normal distribution. EECPS = ethanolic extract of *Carica papaya* seeds; MDA = malondialdehyde; NO = nitric oxide; GSH = glutathione; ANOVA = analysis of variance

Parameter	Control group (oxidative stress)	EECPS-treated group	ISL-treated group	P-value
MDA (nM/mg protein)	4.0	2.0*	1.36*	<0.001
NO (μM/mg protein)	25	15*	16.67*	<0.001
GSH (μM/mg protein)	10	20*	22.3*	<0.001

Dose-response relationship

The reported values for both EECPS and ISL represent an average across the administered doses (400 and 800 mg/kg for EECPS; 20, 40, and 80 mg/kg for ISL). These results indicate a dose-dependent improvement in the antioxidant profile of the treated groups.

Histopathological evaluation

**Figure 3:**
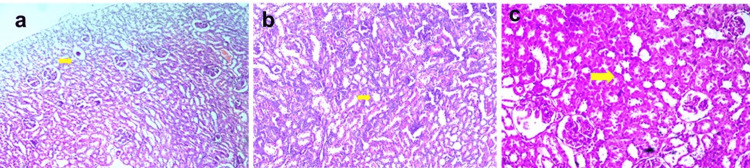
(a) Normal control: Normal renal architecture with no signs of damage or pathological changes. (b) Standard control: Moderate nephrotoxic effects due to silymarin intervention with glomeruli and renal tubules showing signs of partial recovery and lessened damage. Mild to moderate changes in glomerular structure and reduced tubular necrosis reflect the nephroprotective impact of silymarin. Minimal interstitial inflammation and fibrosis indicate a mitigated response to gentamicin-induced injury. (c) Toxicant control: Significant renal damage characterized by glomerular degeneration, pronounced tubular necrosis, and extensive tubular degeneration. There is marked interstitial inflammation and fibrosis (H&E: ×200). H&E = hematoxylin and eosin

In addition to the biochemical assays, histopathological analysis of the kidney tissues provided further insights into the nephroprotective effects of EECPS and ISL. The gentamicin-treated group exhibited marked renal damage, characterized by tubular necrosis, glomerular deterioration, and inflammatory infiltration. Conversely, kidney sections from rats treated with EECPS and ISL displayed significant tissue recovery, with reduced signs of tubular damage, preserved glomerular structure, and decreased inflammatory response (Figure 3).

**Figure 4:**
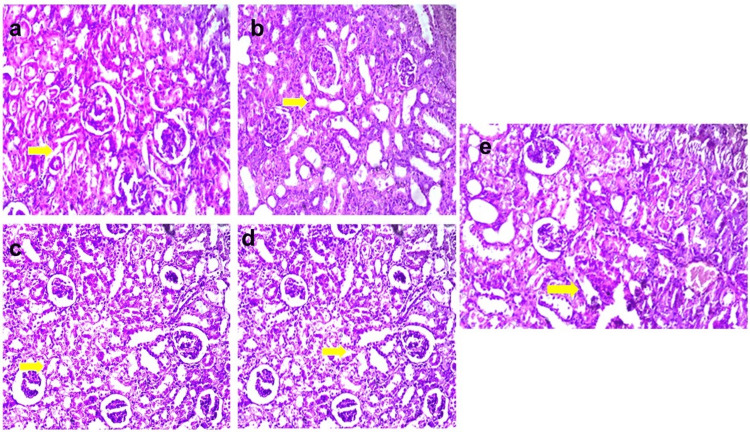
(a) Low-dose EECPS treatment: Renal protection and tissue recovery with reduced tubular necrosis and glomerular damage compared to the toxicant control group. (b) High-dose EECPS treatment: Enhanced renal protection with a significant restoration of kidney architecture and minimal necrosis or damage. (c) ISL 20 mg/kg treatment: Moderate nephroprotection with reduced tubular damage and slight preservation of glomerular structure. (d) ISL 40 mg/kg treatment: Noticeable nephroprotection with improved renal tissue integrity and reduced tubular necrosis. (e) ISL 80 mg/kg treatment: Substantial renal protection with the near-normal restoration of kidney architecture and minimal necrosis or damage (H&E: ×400). EECPS = ethanolic extract of *Carica papaya* seeds; ISL = isoliquiritigenin; H&E = hematoxylin and eosin

These histopathological findings corroborate the biochemical results, illustrating the ability of EECPS and ISL to protect against gentamicin-induced nephrotoxicity (Figure 4).

## Discussion

This study examined the ability of the EECPS and its flavonoid, ISL, to defend against and combat free radicals in Wistar albino rats whose kidneys had suffered damage from gentamicin. The results show that nephrotoxicity markers such as creatinine, urea, and BUN levels dropped significantly after treatment with EECPS and ISL. This supports the idea that these natural compounds can protect against gentamicin-induced kidney damage [11].

When gentamicin was given, serum levels of BUN, urea, and creatinine increased during gentamicin administration. This supports the idea that gentamicin is harmful to the kidneys, as shown in previous research [12]. This elevation is attributed to gentamicin’s capacity to induce oxidative stress and renal inflammation, leading to acute kidney injury [13]. Our findings demonstrated that both EECPS and ISL mitigated these elevations in a dose-dependent manner, with ISL at 80 mg/kg emerging as the most effective dose in reducing creatinine levels. This suggests that ISL has a strong protective effect on the kidneys, which could be because it is an antioxidant and reduces inflammation [14,15].

When compared to silymarin, which is known to protect the kidneys, EECPS and ISL not only worked as well as silymarin but sometimes worked even better. This indicates the substantial therapeutic potential of these natural compounds in managing drug-induced nephrotoxicity [16]. Notably, the fact that these treatments work better or worse depending on the dose suggests an ideal therapeutic window, with ISL at 80 mg/kg showing the best nephroprotective profile [17].

We found that the antioxidant effects were also nephroprotective when we examined oxidative stress markers (MDA, NO, and GSH) in kidney tissue [18]. Following the treatment, there was a big drop in MDA and NO levels and a rise in GSH levels. This is in line with how ISL is known to increase antioxidant effects. These results are in line with other research that has focused on how oxidative stress plays a part in the development of gentamicin-induced nephrotoxicity and how antioxidant therapy can help reduce its effects [18,19].

The fact that ISL was found to be 90.5% pure supports the validity of our results, indicating that the effects we saw are directly related to this bioactive compound. This high purity level highlights the importance of isolating and utilizing high-quality compounds for therapeutic purposes, ensuring the reliability of the nephroprotective and antioxidant effects observed.

Limitations

While this study offers valuable insights into the nephroprotective and antioxidant effects of EECPS and ISL, it has several limitations. First, the research was confined to an animal model, which may not fully replicate the complexity of human renal physiology and pathology. Second, the study focused on acute exposure to gentamicin, not addressing potential long-term effects or chronic kidney disease scenarios. Third, the mechanisms underlying the observed effects were not exhaustively explored, warranting further molecular investigations. Additionally, the study did not compare the efficacy of these natural compounds with a broader range of clinically used nephroprotective agents. Finally, the scalability of extracting and isolating bioactive compounds from *Carica papaya* seeds for therapeutic use remains to be assessed.

## Conclusions

Findings from this study show that EECPS and ISL have a lot of potential to help protect the kidneys and fight free radicals. The identification of a wide range of bioactive substances within EECPS, such as flavonoids, tannins, phenols, alkaloids, proteins, glycosides, and saponins, emphasizes its potency in preventing the nephrotoxicity that gentamicin causes. This study confirms that EECPS and ISL have strong protective and antioxidant effects on the kidneys. This also implies that they could serve as an alternative to or be used alongside gentamicin for treating renal damage induced by its administration.
